# Chronically Implanted Intracranial Electrodes: Tissue Reaction and Electrical Changes

**DOI:** 10.3390/mi9090430

**Published:** 2018-08-25

**Authors:** Andrew Campbell, Chengyuan Wu

**Affiliations:** 1 Sidney Kimmel Medical College, Thomas Jefferson University, Philadelphia, PA 19107, USA; 2 Department of Neurological Surgery, Vickie and Jack Farber Institute for Neuroscience, Thomas Jefferson University Hospital, Philadelphia, PA 19107, USA; chengyuan.wu@jefferson.edu

**Keywords:** intracranial electrodes, foreign body reaction, electrode degradation, glial encapsulation

## Abstract

The brain-electrode interface is arguably one of the most important areas of study in neuroscience today. A stronger foundation in this topic will allow us to probe the architecture of the brain in unprecedented functional detail and augment our ability to intervene in disease states. Over many years, significant progress has been made in this field, but some obstacles have remained elusive—notably preventing glial encapsulation and electrode degradation. In this review, we discuss the tissue response to electrode implantation on acute and chronic timescales, the electrical changes that occur in electrode systems over time, and strategies that are being investigated in order to minimize the tissue response to implantation and maximize functional electrode longevity. We also highlight the current and future clinical applications and relevance of electrode technology.

## 1. Introduction

The brain-electrode interface is one of the most exciting topics in modern neuroscience. Progress in this field represents the culmination of research in separate collaborating disciplines including materials science, neural engineering, and neurosurgery among others. We are now able to record from and stimulate the brain at the level of individual neurons, termed single-units, which holds great promise for future research, and clinical applications.

**Figure 1:**
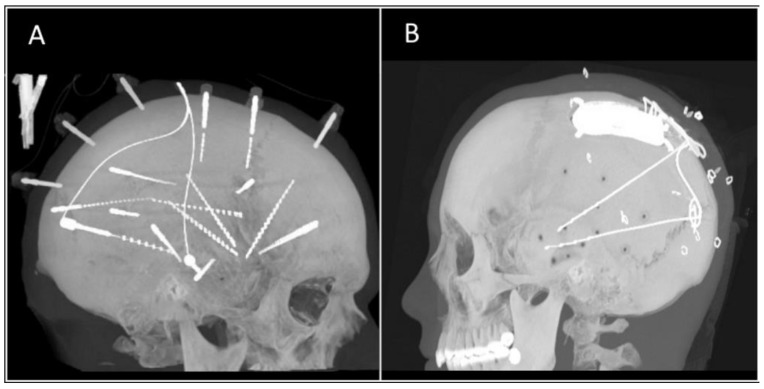
Computed tomography (CT) reconstruction images of patients after implantation of (**A**) sEEG electrodes and (**B**) RNS system.

In humans, advances in recording and neuromodulation technology have led to new medical devices that use electrodes for neural recording, stimulation, or both in the central nervous system (CNS) and peripheral nervous system (PNS). Examples of these breakthroughs include stereo electroencephalography (sEEG), where implanted recording depth electrodes have enhanced our ability to detect and localize epileptogenic foci in the brain (see Figure 1A); deep brain stimulation (DBS), which involves the placement of stimulating electrodes into brain areas for therapeutic effect in conditions like Parkinson’s disease or essential tremor; and responsive neurostimulation (RNS), which aims to both detect seizure activity and respond with electrical stimuli that essentially stops seizures before they start (see Figure 1B). Spinal cord and vagal nerve stimulators as well as cochlear implants are additional examples of neuromodulation devices capable of providing patients relief from back pain, control of drug-resistant epileptic seizures, and even the ability to hear, respectively.

Although these technologies exemplify remarkable medical achievements, further work in this field has involved the incorporation of brain-computer interfaces (BCIs) and neuroprosthetics in humans, which rely on the precision that can be achieved through the implantation of chronic microelectrode arrays into the brain parenchyma. However, there are a number of obstacles that must be overcome before long-term stability of the tissue-electrode interface can be achieved. While the issues of glial encapsulation and electrode degradation remain at the forefront of current thinking, a precise and complete understanding of the mechanisms underlying electrode failure over time has yet to be attained. In this review, we will explore the tissue-electrode interface, with particular focus on the acute and chronic inflammatory reactions, changes that occur in electrodes, and strategies being investigated to mitigate these issues.

## 2. Contemporary Recording and Stimulation Systems 

### Overview

**Figure 2:**
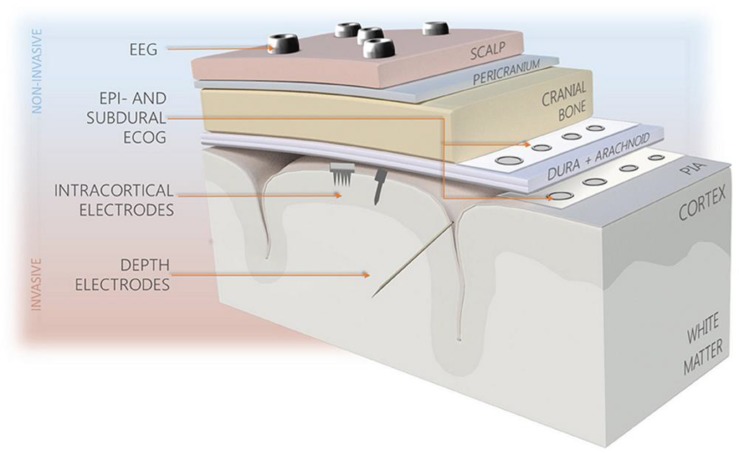
Types of brain interfacing electrodes and their locations in reference to the brain. Reproduced with permission from Creative Commons open access policy from [2].

While a nuanced discussion about electrode hardware is beyond the scope of this review, we will address the basic electrode types in this section. In recent years, there have been numerous electrode systems manufactured for use in scientific and clinical applications. These applications range from recording and stimulation studies in basic neuroscience animal research to deep brain stimulation (DBS) in Parkinson’s disease or intracranial electroencephalography (iEEG) recording for investigating drug-resistant epilepsy. After traditional scalp EEG electrodes, progressively more invasive placement options include epidural, subdural, intracortical, and depth electrodes (see Figure 2). This increased invasiveness puts the electrodes in closer proximity to the neurons producing the signals of interest, which allows for improved detection and stimulation. For example, iEEG using a grid of subdural electrodes, or electrocorticography (ECoG), has distinct advantages over traditional scalp EEG including an increased signal-to-noise ratio, superior spatial resolution, and greater spectral frequency [1]. 

Intracortical electrodes, such as the popular Utah microelectrode array, and depth electrodes are even more invasive and are placed within the brain parenchyma, next to the nuclei or even individual neurons of interest (Figure 2). They allow for an even greater spatial and temporal resolution than subdural electrodes and are capable of single-neuron recording. In contrast with subdural grid placement, depth electrodes in DBS for Parkinson’s disease or sEEG in epilepsy do not require an extensive craniotomy for implantation and can instead be implanted through a small burr hole.

## 3. Tissue Reaction and Histopathologic Observations Due to Electrode Implantation

### 3.1. Overview

Understanding the neural tissue response to device implantation is pivotal for achieving long-term stability and viability of neuroprosthetics and neurostimulation systems. On shorter timescales, recording and stimulating electrodes are able to achieve impressive functionality. But for chronic implants, the body’s response at the tissue-microelectrode interface eventually leads to mechanical failure and signal degradation [3,4,5,6,7,8,9]. Despite extensive investigations into the inflammatory response and numerous trials investigating strategies to mitigate it, clearing this hurdle has remained elusive [5,6,10,11,12,13,14,15,16,17,18,19,20,21,22,23,24,25,26,27,28,29,30,31,32,33,34,35,36,37]. It is consequentially one of the most important obstacles to overcome in order for neural interface technology to be fully realized. In this section we will review current understanding of the tissue reaction to implanted devices.

Microglia, astrocytes, and oligodendrocytes are the three main glial cell types in the brain. The actions of microglia and astrocytes after electrode implantation are key in both short and long-term responses to injury [3]. Microglia function as immunologic surveillance in the CNS by monitoring neuronal health and responding to injury [38]. They function as cytotoxic cells, killing pathogenic organisms and phagocytes that secrete proteolytic enzymes to degrade cellular debris and damaged extracellular matrix during regular turnover or after injury [3]. Microglia exist in an inactivated or “ramified” state, exhibiting a branched morphology, sampling the microenvironment until a stimulus, such as injury, leads to activation [3,38]. Once activated, they begin proliferating and undergo a morphology change to a more compact, “amoeboid” state [3,38]. In this state, the activated microglia phagocytose foreign material, upregulate cell-surface receptors, increase secretion of reactive oxygen species, and produce more lytic enzymes, pro-inflammatory cytokines, and cytotoxic factors [3,38]. Activated microglia have many of the same markers as macrophages, including ED1, which is used for immunohistochemical staining to visualize and quantify microglia in tissue sections [5].

Astrocytes, so named due to their star-like set of cytoplasmic extensions, have many roles in the CNS [3]. These functions include providing mechanical support to circuits of neurons, aiding in control of the neuronal chemical microenvironment, providing growth cues during development, and modulating the firing patterns of neurons [3]. They have a pivotal role in nutrient transfer across the blood brain barrier (BBB), regulating this process via specialized extensions, called end feet, which interface with the capillary walls [3]. After tissue injury, astrocytes become reactive and undergo hypertrophy, proliferation, and upregulate the expression of glial fibrillary acidic protein (GFAP), which is a common target for astrocyte detection in immunostaining [3,5,10,38,39].

Oligodendrocytes have the key role of electrically insulating axons projecting from neurons in the CNS. These cells extend their cytoplasm to form sheaths of myelin that surround axons, greatly increasing the conduction velocity of signals propagating down axons [3].

### 3.2. Initial Injury and Acute Tissue Response to Intraparenchymal Electrode Insertion

When an electrode is inserted into the brain, it first passes through the meninges, consisting of the dura mater (which is usually reflected prior to implantation), the arachnoid, and pia mater. Arteries and veins on the pial surface must be avoided [40]. The cortex below has the highest vascular density in the brain, consisting of numerous capillaries and small-caliber arteries [40]. As the electrode is inserted, these vessels are ruptured, severed, and pulled, leading to bleeding, serum protein leakage, and infiltration of neutrophils, blood-borne macrophages, and T-lymphocytes [3,4,40]. In addition to vascular damage, during insertion, the electrode tears the extracellular matrix, ruptures neuronal and glial cell bodies and processes, and causes tissue displacement [3,4,40]. The electrode pushes progressively more tissue aside as it travels deeper, creating a high-pressure region around it [3]. 

**Figure 3:**
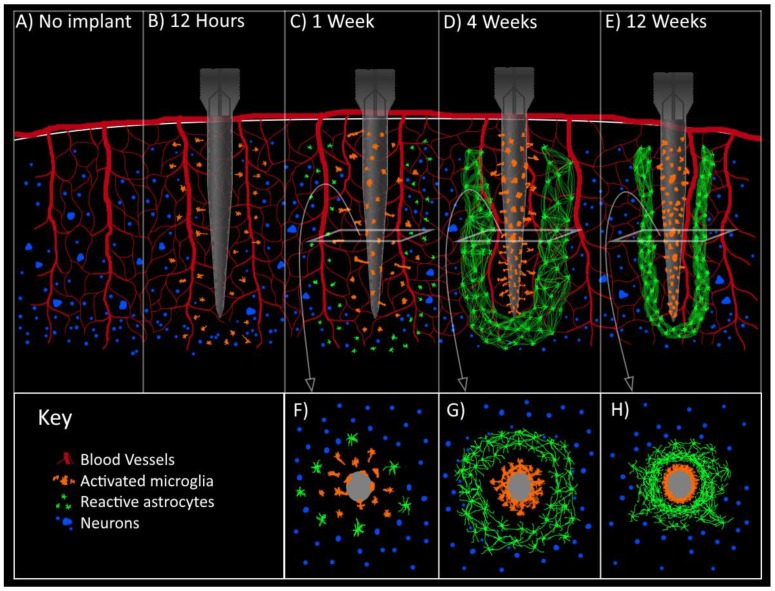
Illustration of the glial encapsulation response (**A**) prior to implantation, (**B**) 12 h post-implantation, (**C**) 1 week post-implantation, (**D**) 4 weeks post-implantation, and (**E**) 12 weeks post-implantation. Panels (**F**), (**G**), and (**H**) represent cross sectional views of (**C**), (**D**), and (**E**), respectively.

Immediately following electrode insertion, microglial cells in the vicinity of the probe become activated, and respond to the injury by sending long projections toward the injury site [11]. Although the mechanism for microglial activation is unknown, the rapidity of this response suggests that a chemical gradient is immediately generated and may be related to cellular debris, messenger molecules released from dying cells, and leaking plasma contents [11,40]. After approximately 12 h, the microglial cells begin to migrate towards the implant; and by 24 h post implantation, the tissue around the implant is surrounded by a greater density of microglial cells (see Figure 3) [4,10,11].

Astrocytes appear to have a more varied and slower reaction than microglia to tissue injury, with a response that becomes robust over several days rather than almost immediately. Results from histochemical analysis and live imaging studies reveal that at least three distinct types of astrocytes react to stab wound injury [10,39]. One study demonstrated significant heterogeneity of astrocyte behavior, where certain subsets of astrocytes proliferated or became polarized and extended processes to the site of injury [39]. It was also shown that astrocytes do not migrate after tissue injury and that increases in detected GFAP immunostaining after injury are due primarily to upregulation of GFAP expression rather than astrocyte proliferation (although some proliferation does occur, particularly in astrocytes close to blood vessels near the injury site) [39].

Release of blood and plasma contents from the disrupted BBB post implantation contributes to the recruitment of activated microglia and astrocyte activation [3,4,6,10,12,40]. Specific plasma proteins that deposit into CNS tissue following vascular disruption include albumin, globulins, fibrin/fibrinogen, thrombin, plasmin, complement, and hemosiderin [4,40]. In particular, thrombin has been implicated in triggering astrogliosis and microglial activation [40]. The cellular effects due to thrombin are mediated by proteinase-activated receptors (PARs) [40]. Thrombin activates the PAR-1 receptor on astrocytes, inducing morphological changes, proliferation, and the release of inflammatory mediators [40]. Mice deficient in PAR-1 were noted to have reduced astrocyte activation after a cortical stab wound, supporting the role of thrombin in astrogliosis [40]. Additionally, thrombin induces microglial proliferation and activation via the action of PAR-1 and PAR-4 receptors, respectively [40]. 

Albumin binds to transforming growth factor beta (TGF-beta) receptors in astrocytes, inducing upregulation of myosin light chain kinase (MLCK) expression [4]. MLCK phosphorylates myosin light chain (MLC), which leads to contractions, weakening of endothelial cell-to-cell adhesion, and ultimately increased BBB permeability [4]. Inhibition of MLCK has been shown to reduce edema following traumatic brain injury in mice, although this did not improve neurologic outcomes or significantly alter lesion histology [41]. Albumin also leads to astrocyte and microglial activation via the mitogen-activated protein kinase (MAPK) pathway, resulting in increased IL-1β and nitric oxide levels in astrocytes [4]. Fibrinogen is polymerized in the perivascular space to fibrin in the CNS leading to the activation of microglia [4]. Depletion of fibrin is associated with inhibition of microglial activation and attenuated inflammatory demyelination of neurons [4].

Many inflammatory mediators are part of the brain tissue response to injury during electrode implantation. Some of the most prominent chemical mediators we will consider are TNF-α, IL-1, IL-6, MCP-1, and TGF-β. TNF-α expression appears to be caused by the initial implantation injury with elevated mRNA levels present around the electrode-tissue interface at one week following implantation that diminish by four weeks [40,42]. Activated macrophages that infiltrate from the breached BBB and activated microglia are significant early sources of TNF-α [40]. IL-1 has many effects on glial cells and neurons, with particularly strong effects on astrocytes, including the promotion and modulation of astrogliosis [40]. IL-1 exists in both a membrane bound (IL-1α) and secreted form (IL-1β) [40]. IL-1β is one of the most significantly upregulated cytokines in the response to implantation, and it is rapidly produced by activated microglia within 15 min of cortical injury [4,40]. It is a major pro-inflammatory cytokine with important roles in inflammation and apoptosis [4].

While TNF-α and IL-1 tend to exert mainly pro-inflammatory effects, IL-6 has both pro-inflammatory and anti-inflammatory properties [40]. IL-6 is produced by microglia, astrocytes, and endothelial cells as part of the downstream sequence of IL-1 and TNF-α signaling, and it has been shown to downregulate the expression of TNF-α and promote neuronal survival and neurite outgrowth [40,43]. Its overexpression causes a pathologic response, leading to reactive gliosis, neurodegeneration, breakdown of the BBB, and angiogenesis [44]. This suggests that tight regulation of IL-6 is necessary in order to promote its beneficial effects [43]. TGF-β is similarly both pro-inflammatory and anti-inflammatory, and it has been shown to inhibit glial cell proliferation as well as expression of IL-1 and TNF-alpha [40,42]. It is normally present in the brain at low levels, but, after injury, appears to be upregulated in astrocytes around the site of tissue damage [40,42]. At these higher concentrations, it plays a role in reactive astrogliosis and scar formation, with studies demonstrating attenuation of the scarring response when TGF-β function is blocked with antibodies [40].

Proteases, such as matrix metalloproteases (MMPs), are expressed in activated glial cells and have been shown to have both beneficial and harmful effects [40]. MMPs may contribute to neuronal cell death by degrading the extracellular matrix protein laminin, but could also facilitate removal of debris after injury via this same process [40]. After a cortical stab wound or electrode implantation, the expression of MMPs is increased within 24 h in the tissue around the site of injury [4,40]. This includes MMP-9 which is known to degrade gap junctions of BBB endothelial cells, and therefore disrupt the BBB [4].

Reactive oxygen species (ROS) are oxygen free radicals that can exert oxidative stress on cells when produced in excess [40]. Following injury, as red blood cells are being broken down, there is an increase in hemoglobin that leads to an increase in ROS [4]. In this context, production of the ROS nitric oxide (NO), which is normally neutralized by cellular antioxidants, can overpower the antioxidants, resulting in cellular damage [39]. In addition, ROS downregulate tight junction proteins, which increases BBB permeability [4]. This setting of increased oxidative stress leads to activation and upregulation pro-inflammatory cytokines [4].

### 3.3. Foreign Body Reaction and Sustained Inflammatory Response

Long-term application of intraparenchymal electrodes is limited partly by a chronic tissue response that consists of the foreign body reaction and sustained inflammation. This leads to the formation of a glial encapsulation or sheath that interferes with the recording and stimulating of neurons. Factors that contribute to this persistent response are initial tissue injury, micromotion of the electrode, persistent BBB leakage, and mechanical compliance mismatch between electrodes and brain tissue. The sustained response appears to be dependent on continual interaction between the electrode and the surrounding tissue. Conversely in stab wound studies, the inflammatory response has been shown to steadily decrease to resolution after the initial injury reaction [13,45]. Similar to the inflammatory response, neuronal cell death that occurs in the space around the electrode also appears to be greatly influenced by the tissue response associated with its continued presence rather than just the initial stab injury [13]. 

One week after implantation, an increased density of ED1 staining microglia can be seen, extending around the injury site covering an area approximately 3–4 times the size of the stab wound void left after electrode explantation (Figure 3) [10]. The ED1 positive microglia closest to the injury site send out short, thick processes, while those further away exhibit a highly elongated morphology [10]. Similarly, an increase in number and intensity of GFAP staining astrocytes is also seen around the wound void [10]. A number of these cells possess cytoplasmic processes that extended to the injury site [10].

At two weeks, the density of ED1 staining microglia immediately around the electrode increases and reactive astrocytes begin forming a GFAP staining sheath that surrounds this space [5,10,13,45]. The cellular distribution appears to represent distinct, segregated layers of ED1 and GFAP staining cells [5,13]. These layers of immunoreactivity suggest a structure characterized by an inner microglial core in contact with the electrode, which drops off by about 50 µm, and a shell of surrounding astrocytes, which predominately occupy the 50–150 µm space surrounding this core [5,10,13]. Neurons in the vicinity of the implant site have died off at this point, indicated by the significantly decreased number of NeuN staining neuronal cell bodies in the 50 µm surrounding the electrode [5,13].

After four weeks, the density of ED1 staining microglia directly around the electrode continues to increase, while the overall amount of ED1 reactivity in the vicinity around the electrode is not significantly different compared to two weeks (Figure 3) [5,13]. The microglia appear to form a more compact structure around the electrode with increasing time [5,13]. In contrast with microglia, statistically significant increases in GFAP reactivity within 100 µm of the electrode at four weeks compared to two weeks have been observed [5]. This suggests that astrocytes play a role in the core region of the glial sheath as the sustained tissue response develops.

At six and 12 weeks, both the ED1 and GFAP staining layers of microglia and astrocytes are more compact and localized (Figure 3) [5,10,45]. Overall the layers become thinner but stronger and denser, with less ED1 and GFAP reactivity found outside of these layers [5,10,45]. This coincides with the peak of GFAP intensity shifting closer to the implant site, suggesting contraction of the reactive astrocytes over time [5]. At this point, the glial sheath has effectively walled the implant off from the rest of the brain tissue with minimal extension of the inflammatory response into the surrounding area. It should be noted that at these later time points, there is significant variability in the degree of these immunohistochemically visualized cellular responses, which tend to coincide with similar variability in the changes occurring in individual electrodes [5,46,47].

### 3.4. Tissue Response to Subdural Electrodes

While the above discussion has been focused mainly on the tissue response to intraparenchymal electrode insertion, a chronic foreign body reaction occurs due to subdural electrode implantation as well. There is a growing interest in the use of chronic subdural electrodes in brain machine interfaces and neuroprosthetics; but, to the best of our knowledge, there is a paucity of literature describing the chronic inflammatory changes associated with subdural ECoG systems [48]. Therefore, long-term studies evaluating tissue response and subdural electrode function are necessary to determine viability and feasibility for extended use. 

Histological studies that have been conducted thus far in humans involve shorter timescales, as they are limited to patients receiving invasive monitoring for epilepsy who subsequently undergo surgical resections. In one study, tissue samples were examined on the order of days to several weeks after subdural electrode implantation, and have shown chronic meningeal and perivascular inflammation consisting of lymphocytes and macrophages to be the most common histopathologic finding [49]. Longer investigations have been performed in animals, with recent studies involving the use of micro-ECoG subdural implants in rats revealing vascular growth through holes in the electrode substrate material and moderate tissue reactivity at 25 weeks post implantation on histopathologic analysis [50,51]. Similarly, a nearly two-year case study of a rhesus monkey implanted with a subdural ECoG array demonstrated minimal inflammatory response, with fibrous encapsulation surrounding the grid following explantation [48]. The cortex below the implant appeared to be unaffected by the grid and was consistent with the contralateral hemisphere where no grid had been placed [48]. These results, as well as the success of long term RNS systems in human patients, are encouraging for the prolonged application of subdural electrodes.

## 4. Changes in Electrode Signaling Over Time

### Factors Influencing Electrode Function

There are many factors that contribute to the changing function of electrodes. With time, the common endpoint of these changes is a diminished ability to record neurons and elicit responses through stimulation [4,6,7,8]. The metric most often correlated with changes in electrode function is electrical impedance at the tissue-electrode interface. Impedance refers to the resistance to the flow of charged particles, or current, in a circuit or circuit component. At the tissue-electrode interface, impedance is inversely related to the volume of tissue activated by stimulating electrodes and the listening sphere of recording electrodes [52,53]. If the impedance is low, current can flow more freely in the volume around the tissue-electrode interface, resulting in a larger stimulation and recording radius. In addition to measurement of the magnitude of impedance, some groups have used complex impedance spectroscopy as a method of detecting more specific changes at the tissue-electrode interface [46,47,54,55].

In the first few weeks after implantation, impedance rises quickly and then begins to stabilize [47,54,55,56]. The initial rise is thought to be due to the tissue reaction and this is supported by histological studies correlating the degree of tissue encapsulation with the magnitude of impedance increases [47,55]. Glial scarring and the attachment of molecular and cellular species to the electrode surface increase impedance, but these components are not considered to be sufficient to hinder electrode function or cause failure [56,57]. This finding has led some groups to suggest that eliminating the glial scar may not be required in order to achieve long term electrode stability [56].

**Figure 4:**
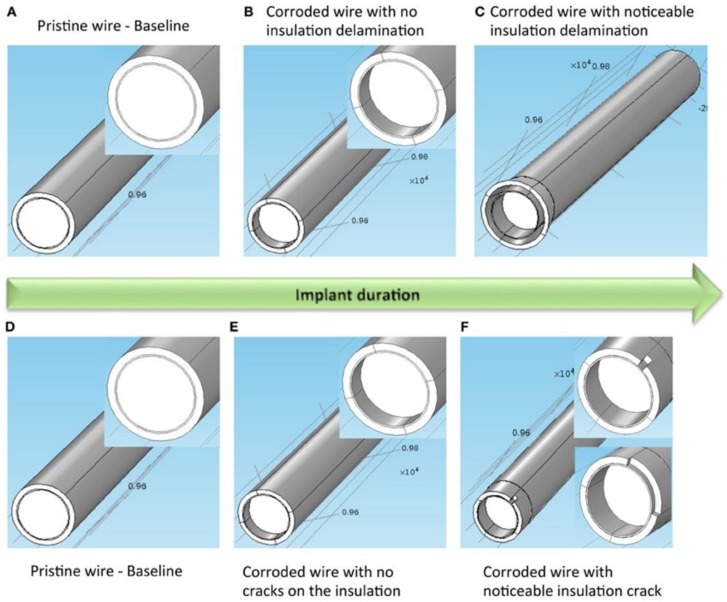
Two models of electrode degradation. (**A**) Pristine electrode with intact metal and insulation, (**B**) electrode with corroded metal and no insulation delamination, and (**C**) electrode with corroded metal and noticeable insulation delamination. (**D**) Pristine electrode with intact metal and insulation, (**E**) electrode with corroded metal and no insulation crack, and (**F**) electrode with corroded metal and noticeable insulation crack. Inset on the left, middle and right images shows a closer view of the gold layer around the tungsten. Illustration and caption reproduced with permission from Creative Commons open access policy from [58].

While the precise interplay is not currently understood, several influences may contribute to the impedance changes that occur at the tissue-electrode interface. These factors can be roughly differentiated by biotic effects and abiotic effects [53,58,59]. Biotic effects refer to tissue reactions such as glial encapsulation, BBB disruption, and macrophage recruitment to the implant site [53,58,59]. Abiotic effects refer to causes of electrode degradation including electrode corrosion, insulation delamination, and cracking (See Figure 4) [53,58,59]. Insulation delamination of the electrode, caused by the corrosion and degradation secondary to the persistent inflammatory response, can result in a lower impedance than the original reference impedance of an electrode [58,59].

In a long term study in non-human primates, one group showed that, for silicon-based intracortical microelectrode arrays (MEAs), the most common type of electrode failure was acute mechanical failures, accounting for 48% percent of the failures in the study, while biological causes accounted for about 24% of failures [8]. They found that most failures occurred in the first year after implantation and that, after an initial increase, impedance slowly declined along with signal quality, suggesting that insulation material degradation is one of the most important factors in long term viability of silicon MEAs [8]. This same group also performed scanning electron microscopy (SEM) on silicon MEAs with platinum electrode tips implanted for long durations in non-human primates, and revealed that progressive corrosion occurs over time at the electrode tips as well as cracking and delamination of the parylene insulation [9]. In addition, the tissue encapsulation response was shown to grow into defects formed in the platinum and parylene over time [9].

Interestingly, clinically-relevant stimulation in DBS electrodes has been associated with rapid and reversible changes in impedance [54]. Immediately following the cessation of stimulation, impedance begins to rise and eventually reaches pre-stimulation levels within a few days, revealing the dynamic nature of the foreign body reaction [54]. It is thought that stimulation temporarily “cleans off” adherent molecules and cells that are attached to the electrode, but it does not clear away the encapsulation sheath surrounding the electrode [54,60]. This “rejuvenation” stimulation has also been shown to decrease impedance and increase the signal to noise ratio at the electrode-tissue interface, and it has been suggested that this could be used as treatment to improve the quality of stimulation and recording in chronically implanted microelectrodes [60]. As such, both short term and long term factors appear to effect electrode impedance [53]. Electrode functionality can be predicted with impedance measurements, and actively controlling impedance over time may be a possible strategy to improve long term performance and neuronal yield [53].

## 5. Strategies for Reducing Chronic Tissue Reaction

### 5.1. Overview

Many strategies to reduce the foreign body reaction have been investigated that focus on different factors involved in the generation of the response. The main types of approaches can be broken down into mechanically based and biologically based, with some degree of overlap existing. These strategies range from adjusting material properties of the electrodes to delivering anti-inflammatory drugs to the implant site. Minimizing neuronal loss, promoting neural regeneration, and limiting the formation of the glial sheath are the end goals of these different strategies. A combined approach will likely be necessary due to the multiple mechanisms involved in the inflammatory and regenerative responses.

### 5.2. Insertional Approaches

If we start from the beginning of the electrode implantation process, planning the insertional trajectory is one way to potentially reduce the tissue response. Avoiding damage to major blood vessels can lessen the overall BBB disruption, minimizing this contribution to the overall post-implantation inflammation [12]. Large intracortical blood vessels do not penetrate perpendicularly into the brain, but instead deviate slightly from the normal axis [12]. Inserting electrodes on the normal axis at a distance greater than 49 µm from a major blood vessel greatly reduces the likelihood of disrupting the penetrating segment of these vessels deeper in the brain [12]. Interestingly, neuronal nuclei lie further away from blood vessels than would be expected in a random distribution, which suggests that avoiding major blood vessels would also allow for better recording and stimulation [12]. In addition to trajectory, the insertional velocity and implant diameter has also been studied, with evidence showing fast insertions (2000 µm/s) and sharp implants to be superior at minimizing tissue strain and vascular injury [14].

### 5.3. Mechanical Approaches

Mechanical considerations such as the size, flexibility, and material density of electrodes relative to the tissue density are important factors in the foreign body reaction. Compliant electrodes can be made using ultra-thin geometries or very soft materials [15]. Newer generations of electrodes have become considerably smaller and thinner, and it has been shown that smaller implants lead to less initial tissue damage, decreased neuronal loss in the vicinity of the electrode, and reduced chronic tissue responses [15,16]. Similarly, implanted ultrasoft microwire electrodes consisting of elastomers and conducting polymers mechanically similar to brain tissue display a reduced inflammatory response compared to tungsten electrodes [17].

The thinking behind flexible and density matched electrodes is that they exhibit less micromotion related trauma and inflammation over time and therefore increase electrode longevity. Flexible microwire electrodes implanted into rabbit cortex displayed diminished foreign body response and increased neuronal density around the electrode relative to conventional microwire over 26–96 weeks indwelling periods [18]. This is despite the insertional method for the flexible microwire electrode being inherently more traumatic than conventional microwire, due to the requirement of a stiff, sharp carrier to guide it into place [18]. The densities of stainless steel (8 g/cm^3^), tungsten (19.25 g/cm^3^), and platinum (21.45 g/cm^3^) and iridium (22.6 g/cm^3^) are much greater than that of brain tissue (0.99 g/cm^3^) [19]. Significantly reduced astrocytic and microglial reactions have been observed at 6 weeks post implantation around 500 µm diameter low density electrodes more analogous to that of brain tissue (1.16–1.48 g/cm^3^) relative to high density electrodes of the same size (22.45 g/cm^3^) [19].

In one study, mechanically-adaptive implants were introduced into the brain that were rigid at first but became compliant after implantation [20]. Interestingly, while the acute tissue response was comparable to stiff control implants, the chronic inflammatory response was significantly reduced and the BBB was more stable [20]. Another interesting strategy being investigated involves promoting integration of the surrounding brain tissue with electrodes by structuring the surface topography of electrodes on the microscale or nanoscale level, with some results indicating increased neuronal survival in the 100 µm surrounding the implant [21].

Of note, it has been shown that implants tethered to the skull have a significantly increased inflammatory response [22]. This is thought to be due to the migration and colonization of fibroblasts from the meninges to the electrode-tissue interface [22]. Finally, these tissue reactions appear to occur independently; when multiple electrodes were separately implanted onto rat cortex, they did not aggravate the tissue reactions occurring at the other implant sites [23].

### 5.4. Biological Approaches

Biologically based strategies focus on attenuating the inflammatory response and promoting neuronal regeneration. Dexamethasone, a synthetic glucocorticoid, has been infused intramuscularly, coated on electrodes, and perfused through microdialysis probes, with all methods diminishing the reactive tissue response [24,25,26]. Probes coated with neural adhesion molecule L1 have been shown to have a reduced early microglial response, completely diminished loss of neuronal cell bodies, increased axonal density in the electrode vicinity relative to the background tissue, and significantly lowered activation of microglia and reactive astrocytes relative to uncoated probes [27,28]. A recent study showed that an astrocyte-derived extracellular matrix coating reduced the degree of astrogliosis surrounding a chronically implanted electrode [29].

Peptide-based coatings are also being explored with promising preliminary in vitro results [30]. In addition, silicon-based probes seeded with neural progenitor cells have been implanted into rat brains with early results showing that the cells can be successfully be implanted and may diminish the surrounding astrocytic response [31]. Probes capable of both drug delivery and electrophysiology recordings have also been designed and implanted with early success [32].

Targeting CD14 in circulating myeloid cells and not brain-derived microglia has been shown to improve chronic microelectrode recording, measured by the number of single neuron units detected per electrode channel and the percentage of electrode channels detecting single neurons [33]. The role of CD14 activity in microglia may be neuroprotective as complete knockout of CD14 in mice did not produce as a strong an improvement in microelectrode recordings as CD14 inhibition in macrophages [33]. This group also demonstrated that complete knockout of CD14 resulted in microelectrode improvement in acute but not chronic time periods, while inhibition of CD14 using a small molecule inhibitor called IAXO-101 improved both acute and chronic performance [34]. Together, these results suggest that therapies do not necessarily need to cross the BBB to benefit the quality of microelectrode recording after implantation [33,34].

Anti-oxidant therapy using resveratrol has been investigated as a potential therapeutic agent for mitigating the inflammatory response to electrode implantation [35,36,37]. Resveratrol is an anti-oxidant molecule derived from grapes that can suppress the accumulation of ROS [35]. In one study, resveratrol was incorporated onto intracortical implants consisting of physiologically responsive mechanically adaptive nanocomposites [35]. The materials in this device are initially stiff, allowing for placement, but then soften after implantation, becoming mechanically compliant with the brain tissue [35]. This material combined with the film of resveratrol, allowing for three days of localized delivery, resulted in reduced microglial activation and improved neuronal density at two weeks post implantation [35]. Another group studied the effects of intraperitoneal resveratrol injections in rats administered both 16–24 h before and immediately following implantation of single-shank Michigan style electrodes [36,37]. The results suggest that initial suppression of reactive oxygen species leads to chronic improvement in neuronal viability [36,37]. Decreased expression of toll-like receptor 4 was identified at week 2 post implantation, but not at later time points, and likely contributes to these beneficial effects [36]. However, intraperitoneal administration of resveratrol was also associated with some side effects in this study, including increased BBB permeability and adhesions [37].

## 6. Conclusions

The current progress toward achieving stable, long term electrode implantation is promising, but additional improvements still need to be made. A complete understanding of the tissue response to electrodes and mastery of implant materials and biocompatibility will enhance our ability to manipulate the foreign body reaction and lead to electrodes that do not degrade over time, allowing for the realization of chronic viability. These are challenging problems that have been investigated for decades, testing the creativity and intuition of several scientific and engineering disciplines, but incremental progress has been made and is poised to continue.
